# A longitudinal study of patients with cirrhosis treated with L-ornithine L-aspartate, examined with magnetization transfer, diffusion-weighted imaging and magnetic resonance spectroscopy

**DOI:** 10.1007/s11011-016-9881-3

**Published:** 2016-08-03

**Authors:** Vijay P. B. Grover, Mark J. W. McPhail, Marzena Wylezinska-Arridge, Mary M. E. Crossey, Julie A. Fitzpatrick, Louise Southern, Brian K. Saxby, Nicola A. Cook, I. Jane Cox, Adam D. Waldman, Novraj S. Dhanjal, Aluel Bak-Bol, Roger Williams, Marsha Y. Morgan, Simon D. Taylor-Robinson

**Affiliations:** 1 Liver Unit, Division of Digestive Health, Department of Surgery and Cancer, Imperial College London, 10th Floor QEQM Wing, St Mary’s Hospital, Praed Street, London, W2 1NY UK; 2 Robert Steiner MRI Unit, Imaging Sciences Department, MRC Clinical Sciences Centre, Imperial College London, London, UK; 3 Centre for Ageing and Health, Newcastle University, Newcastle-upon-Tyne, UK; 4 The Foundation for Liver Research, Institute of Hepatology, 69-75 Chenies Mews, London, WC1E 6HX UK; 5 Division of Brain Sciences, Hammersmith Hospital Campus, Imperial College London, London, UK; 6 UCL Institute for Liver & Digestive Health, Division of Medicine, Royal Free Campus, University College London, London, UK

**Keywords:** Cirrhosis, Hepatic encephalopathy, Magnetic resonance imaging, Spectroscopy

## Abstract

The presence of overt hepatic encephalopathy (HE) is associated with structural, metabolic and functional changes in the brain discernible by use of a variety of magnetic resonance (MR) techniques. The changes in patients with minimal HE are less well documented. Twenty-two patients with well-compensated cirrhosis, seven of whom had minimal HE, were examined with cerebral 3 Tesla MR techniques, including T_1_- and T_2_-weighted, magnetization transfer and diffusion-weighted imaging and proton magnetic resonance spectroscopy sequences. Studies were repeated after a 4-week course of oral L-ornithine L-aspartate (LOLA). Results were compared with data obtained from 22 aged-matched healthy controls. There was no difference in mean total brain volume between patients and controls at baseline. Mean cerebral magnetization transfer ratios were significantly reduced in the globus pallidus and thalamus in the patients with cirrhosis irrespective of neuropsychiatric status; the mean ratio was significantly reduced in the frontal white matter in patients with minimal HE compared with healthy controls but not when compared with their unimpaired counterparts. There were no significant differences in either the median apparent diffusion coefficients or the mean fractional anisotropy, calculated from the diffusion-weighted imaging, or in the mean basal ganglia metabolite ratios between patients and controls. Psychometric performance improved in 50 % of patients with minimal HE following LOLA, but no significant changes were observed in brain volumes, cerebral magnetization transfer ratios, the diffusion weighted imaging variables or the cerebral metabolite ratios. MR variables, as applied in this study, do not identify patients with minimal HE, nor do they reflect changes in psychometric performance following LOLA.

**Electronic supplementary material:**

The online version of this article (doi:10.1007/s11011-016-9881-3) contains supplementary material, which is available to authorized users.

## Introduction

It is widely accepted that the presence of minimal hepatic encephalopathy (HE) has negative effects on quality of life and survival (Weissenborn 2015). However, there is no universally-accepted, robust and objective measure for determining its presence. The absence of such a measure is likely to hamper the progress of therapeutic trials of medications that may be of benefit to people with this condition. At present, the Psychometric Hepatic Encephalopathy Score (PHES) battery (Weissenborn et al. 2001) is considered to provide the best clinical standard for evaluating cognitive function in patients with cirrhosis, although testing can be time-consuming and language-specific normative data are not available for all population subsets.

Cerebral magnetic resonance imaging (MRI) is a useful tool for non-invasively assessing not only brain structure, but also provides measures of cerebral volume and brain water content. The techniques which have been utilized in patient with HE include:

(i) Volumetric measurements: these have been applied in a variety of clinical contexts including both minimal and overt HE (McPhail et al. 2013; Patel et al. 2004).
(ii) Magnetization transfer imaging: several investigators have reported reduced magnetisation transfer ratios (MTR) in various regions of the brain in patients with cirrhosis (Balata et al. 2003; Iwasa et al. 1999; Rai et al. 2015; Rovira et al. 2001; Taylor-Robinson et al. 1995). Proposed mechanisms for this change in brain tissue characteristics include alterations in astrocyte membrane permeability and cytoplasmic structure and subsequent shifts in the distribution of macromolecules and intracellular water with an element of both subtle intracellular oedema and an increase in extracellular fluid (Grover et al. 2006). The reduction in MTR may reverse after liver transplantation (Córdoba et al. 2001) and after treatment for HE with rifaximin and lactulose (Rai et al. 2015), but there is no definitive evidence that the changes observed relate to the presence or severity of HE.
(iii) Diffusion-weighted imaging (DWI): this allows non-invasive probing of tissue ultrastructure, by quantifying movement of water molecules. Two quantitative measures are derived from the diffusion data, namely the apparent diffusion coefficient (ADC) and the fractional anisotropy (FA).

The ADC is a measure of tissue water diffusivity; an increase in ADC is regarded as indicating vasogenic or extracellular oedema (Ebisu et al. 1993; Schaefer et al. 2000; Schwartz et al. 1998), while a reduction in ADC is interpreted as representing cytotoxic or cellular oedema (Sotak 2004; van der Toorn et al. 1996). Studies in patients with cirrhosis and varying degrees of HE have reported increased regional cerebral ADCs (Lodi et al. 2004; Sugimoto et al. 2008), with a degree of reversibility observed following treatment for HE (Ahluwalia et al. 2014; Chen et al. 2015; Kale et al. 2006). These findings have been attributed to: (i) increased brain water content (Lodi et al. 2004); (ii) reversible interstitial oedema (Kale et al. 2006); (iii) minimal cellular oedema with an increase in membrane permeability and intracellular diffusivity, and changes in the viscosity of the cytoplasm (Sugimoto et al. 2008).

Fractional anisotropy (FA) is a measure of the overall directionality of water diffusion. Chen et al. (2015) reported significant *reductions* in mean FA throughout the cerebral white matter in patients with minimal HE, compared to their neuropsychiatrically unimpaired counterparts, but data from healthy controls were not included for comparison. Ahluwalia et al. (2015), reported significant *increases* in mean FA in patents with minimal HE following 2 months of treatment with rifaximin; however, no healthy controls were included and so the FA data before treatment are not easily interpreted. Lin and colleagues found no significant changes in mean FA in 28 liver transplant candidates with minimal HE compared with 30 healthy matched controls (Lin et al. 2014).

A reduction in FA in the corpus callosum has been reported in people with a history of alcohol abuse, without apparent liver disease, indicating that this may be an important confounder (Pfefferbaum et al. 2006; Pfefferbaum and Sullivan 2002), although the majority of patients included in the present study and in the others reported to date had non-alcohol-related cirrhosis.

Cerebral proton magnetic resonance spectroscopy (^1^H MRS) can be used to determine the biochemical profile of an area of interest non-invasively. Several investigators have reported characteristic spectral appearances in patients with HE, including a reduction in the myo-inositol (mI) and choline (Cho) resonances and an increase in the glutamine/glutamate composite (GLx) resonance (Córdoba et al. 2001; Häussinger et al. 1994; Laubenberger et al. 1997; Miese et al. 2006; Ross et al. 1992; Taylor-Robinson et al. 1994, 1996). The generally accepted explanation for these findings is that intracellular osmolytes, such as mI, are expelled from astrocytes in order to limit the degree of cell swelling which results from the influx of excess ammonia and the subsequent accumulation of osmotically active glutamine (Taylor-Robinson et al. 1996).

Although there is some evidence that cerebral MR might be useful to detect minimal HE (Chen et al. 2015; Rai et al. 2015) and to determine the effects of treatment (Ahluwalia et al. 2014; Rai et al. 2015), relatively few well-controlled studies are available, to date. The aim of this study was to determine if measures of cerebral volume, water content and metabolite concentrations, obtained using cerebral MR imaging and spectroscopy, can be used to differentiate patients with minimal HE from their neuropsychiatrically unimpaired counterparts and/or to monitor treatment effects.

Consecutive patients with cirrhosis with no current evidence or past history of HE and a carefully matched healthy control population underwent a combination of cerebral MR techniques including:

1. MT to derive quantitative measures of brain water content/membrane fluidity;
2. DWI to assess intracellular and extracellular water and structural integrity;
3. Volumetric imaging to determine brain volume;
4. ^1^H MRS to delineate the brain metabolite profiles.

Patients were reassessed and MR studies repeated in the patients with cirrhosis after treatment for 4 weeks with oral L-ornithine L-aspartate (LOLA), a specific ammonia lowering agent.

## Methods

### Subjects

The patient population comprised of 22 right-handed individuals (15 men: 7 women; mean age [range], 51 [37–65] years) with biopsy-proven cirrhosis, recruited sequentially from amongst those attending liver outpatient clinics at the Imperial College Healthcare Trust, London. The aetiology of the cirrhosis was alcohol in five (23 %); hepatitis C in five (23 %); alcohol and hepatitis C in four (18 %); primary biliary cirrhosis in three (14 %); autoimmune hepatitis and haemochromatosis in two each (9 %) and hepatitis B in one (5 %); all patients with alcohol–related liver disease had been abstinent from alcohol for a minimum of 6 months before participating. All 22 patient had well-compensated Child-Pugh Grade A cirrhosis and were clinically stable at the time of enrolment with no clinical evidence of neuropsychiatric impairment. Patients were excluded from the study if they had a history of cerebrovascular disease or of major psychoses, type I diabetes, or type II diabetes with macrovascular complications, renal impairment, hyponatraemia (serum sodium <130 mmol/L), decompensated cardio-respiratory function, or were currently using illicit intravenous drug or taking prescribed psychoactive medication. They were also excluded if their manual dexterity was impaired or if they could not speak English. None had contraindications to MRI scanning.

A total of 22, age-matched, right-handed, healthy volunteers (10 men: 12 women; mean age 47 [36–64] years) were recruited from amongst visitors attending and staff working in Imperial College Healthcare Trust. The same exclusion criteria were applied when recruiting controls as when recruiting the patients. In addition, individuals were excluded if they drank alcohol in excess of National guidelines (16 g/day for women and 24 g/day for men) or if they were taking prescribed or over the counter medications. None had contraindications to MRI scanning.

### Overall study design

All patients were comprehensively assessed at baseline in a single session, lasting approximately 3 h, during which they were clinically examined, blood was drawn for routine haematology and chemistry and they underwent psychometric testing and comprehensive MR scanning. Patients were then prescribed 9 g/day of oral L-ornithine L-aspartate, in divided doses, for 28 days following which the same procedures were repeated.

The healthy controls underwent MR scanning using an identical procedure to that adopted in the patients; scanning was repeated in eight of the 22 controls after a median (range) of 52 (46–56) days.

#### Psychometric testing

Psychometric testing was undertaken, by one of two individuals (MMEC and VPBG), in a quiet room with constant light level using the PHES battery (Weissenborn et al. 2001), which comprises of five paper and pencil tests viz.: digit symbol, number connection A and B, serial dotting and line tracing. The PHES data were adjusted and scored using UK normative data (Marks et al. 2008); composite scores of less than two standard deviations below mean reference values were considered abnormal.

#### MR imaging: data acquisition and analysis

Cerebral MRI was performed on a 3 T Philips Intera™ MR system (Philips, Best, The Netherlands). Standard volumetric imaging was performed using a T_1_-weighted three-dimensional (3D) imaging sequence with the following acquisition parameters: repetition time (TR) 256 ms, echo time (TE) 3.8 ms, number of signal averages (NSA) 1, 256 image matrix, 25 cm field of view (FOV) and 2.0 mm slice thickness. T_2_-weighted sequences were performed to exclude structural brain pathology, with the following sequence parameters: TR 3000 ms, TE 80 ms, NSA 2, image matrix of 230, 23 cm FOV, and 3.0 mm slice thickness. DWI was obtained in 15 directions of sensitization using single-shot echo planar imaging with the following sequence parameters: TR 12555 ms, TE 51 ms, slice thickness 2 mm, NSA 2, b1000s/mm^2^. A SENSE factor of 2 was used to reduce image distortion. MT was obtained using a two-dimensional gradient-echo pulse sequence with the following parameters: TR 54.7 ms, TE 3.75 ms, flip angle 15 degrees, slice thickness 2 mm, 1 NSA with 20 slices positioned over the basal ganglia.

Brain volumes were calculated in FMRIB software library (FSL v4.1, University of Oxford, UK), using SIENAX (www.fsl.fmrib.ox.ac.uk/fsl/fslwiki/SIENA) following guidance on calculation algorithms provided by Klauschen et al. (2009). Co-registration using the exterior skull surface to align images prior to and following treatment was performed using the T_1_-weighted images. The skull was then removed from the images using the brain extraction tool (BET) within the software package. Tissue class segmentation was then performed on the images to calculate the total grey and white matter volumes (excluding CSF). The fractional intensity threshold was set to the default value of 0.35 and standard brain masking was utilised.

MTR maps were calculated, using ImageJ® version 1.32j, (www.imagej.nih.gov) using the formula:

$$ MTR=100\left({SI}_0-{SI}_{RF}\right)/{SI}_0, $$

where *SI*_*RF*_ is the signal intensity in the image employing an off-resonance *RF* pulse and SI0 the signal intensity in the initial proton density image.

Regions of interest (ROIs) were drawn around: (i) the frontal white matter; (ii) the head of the caudate; (iii) the putamen; (iv) the globus pallidus; and, (v) the thalamus bilaterally. Standardized areas within the ROIs were used for comparisons of data between subjects.

ADC and FA maps were calculated from the DWI data sets using DTI Studio version 2.1 (www.dsi-studio.labsolver.org). ADC and FA values were recorded from specific ROIs in the genu, body and splenium of the corpus callosum. These areas were chosen as they were anatomically highly conspicuous and hence easily defined on this imaging sequence. Standardized areas within the ROIs were used for comparisons of data between subjects.

#### MR spectroscopy: data acquisition and analysis

^1^H MRS was acquired using a SENSE head coil and a short echo time PRESS sequence with the following parameters: TR 2000 ms, TE 36 ms, NSA 64, with volumes of interest of 15 × 15 × 15 mm placed in the left basal ganglia. The sequence was performed three times to give a total NSA of 192.

^1^H MR spectra were analyzed by two observers (MW and LS), blinded to the clinical status of the subjects. Peak areas were measured for choline-containing compounds (Cho), creatine/phosphocreatine (Cr), myo-inositol (mI), glutamine/glutamate (Glx) and N-acetylaspartate (NAA), using the ‘Advanced Method for Accurate, Robust and Efficient Spectral Fitting of MRS Data’ (AMARES) algorithm, included in the jmrui software package (www.jmrui.eu), in the time domain. Peak area ratios for NAA/Cr, Cho/Cr, Glx/Cr and mI/Cr were then calculated as previously reported (McPhail et al. 2013).

### Statistical methods

Data were tested for normality using the Shapiro-Wilk test. Between-group comparisons were made with the Mann-Whitney U test or Kruskall Wallis test with Dunn’s correction for multiple comparisons. Longitudinal data were analysed using the Wilcoxon signed rank sum test. Correlations were made with the Spearman rank test. Tests of significance were two-tailed. Statistical analyses were performed using SPSS version 16 (SPSS Inc., USA). Where multiple brain regions were analyzed, a multiple correction factor of n-1 was applied. The relationships between continuous MR variables (MTR, ADC and spectral intensities) were examined using Pearson’s *r* with a false discovery rate modification of the *p* value for significance of <0.005.

### Ethics

Ethical approval was obtained from the joint Hammersmith Hospital/Queen Charlotte’s & Chelsea Hospital Research Ethics Committee (ref 04/Q0406/161), London, UK. Local Research Governance approval and indemnity was provided by Imperial College London. All subjects provided written informed consent.

## Results

### Baseline psychometric status

At baseline, seven (32 %) of the 22 patients with cirrhosis were classified as having minimal HE, based on the results of the PHES test battery, while the remaining 15 patients were classified as neuropsychiatrically unimpaired.

### Baseline MR variables

Baseline cerebral imaging variables were available in 22 patients and 22 control subjects. MRS variables were available in 20 patients and 22 controls.

#### Brain volume

There was no significant difference in total brain volume between patients and healthy controls.

#### Magnetization transfer ratios

**Fig. 1:**
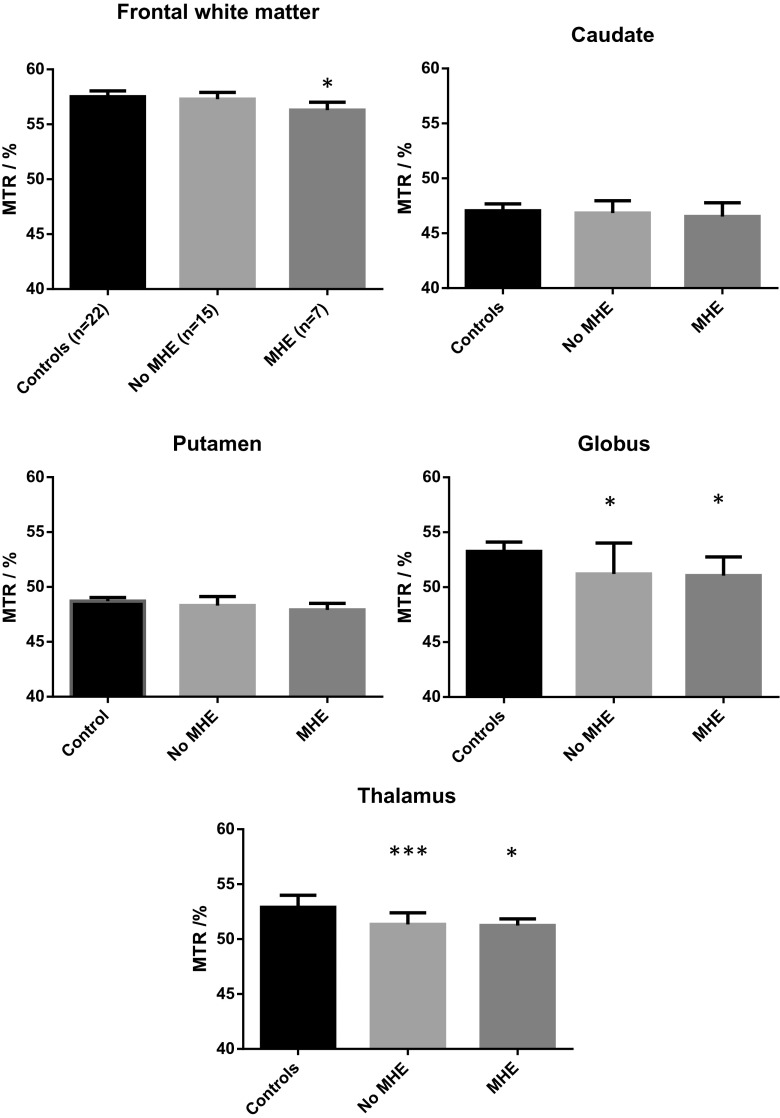
Regional cerebral Magnetisation Transfer Ratios in healthy controls and in patients with cirrhosis, by neuropsychiatric status. Data are presented as median (IQR). Significance of the difference from healthy controls *p < 0.05, **p < 0.01, ***p < 0.001

Mean MTRs were significantly reduced in the frontal white matter (1.8 %), globus pallidus (3.7 %) and thalamus (2.1 %) in the patients with cirrhosis, compared to the healthy controls (Table 1). There were no differences in the reductions in MTRs in the globus pallidus and thalamus in relation to neuropsychiatric status, whereas the reduction in mean frontal white matter MTR was only observed in the patients with minimal HE (Fig. 1). However, there was no significant difference between frontal white matter MTR in the patients with minimal HE and their unimpaired counterparts.

**Table 1:** Baseline MR imaging and spectroscopy variables in patients with cirrhosis and healthy controls

MR Variable	Healthy controls (*n* = 22)	Patients with cirrhosis (*n* = 22)	Significance (*p*)
Magnetization Transfer Ratio			
Frontal WM	57.5 (57.0–58.0)	57.0 (55.7–57.4)	**0** **.023**
Caudate	47.0 (46.6–47.6)	46.8 (45.7–47.6)	0.545
Putamen	48.7 (47.9–49.0)	48.2 (47.3–48.9)	0.147
Globus pallidus	53.4 (52.9–53.7)	51.4 (50.5–52.6)	**0.001**
Thalamus	53.0 (52.3–53.5)	51.5 (50.5–51.9)	**<0.001**
DWI: Corpus Callosum: ADC (10^–3^ mm^2^/s)			
Genu	0.83 (0.79–0.87)	0.82 (0.808–0.843)	0.763
Splenium	0.72 (0.70–0.75)	0.72 (0.71–0.77)	0.951
Body	0.80 (0.75–0.82)	0.83 (0.80–0.84)	0.088
DWI: Corpus Callosum: FA			
Genu	0.74 ± 0.05	0.75 ± 0.03	0.647
Splenium	0.86 ± 0.06	0.85 ± 0.04	0.608
Body	0.73 ± .0.03	0.75 ± 0.04	0.051
^1^H-MRS Left Basal Ganglia			
NAA/Cr	1.30 ± 0.21	1.55 ± 0.51	0.096
Cho/Cr	0.81 ± 0.17	0.79 ± 0.23	0.745
mI/Cr	0.35 ± 0.11	0.42 ± 0.21	0.173
Glx/Cr	0.77 ± 0.28	1.05 ± 0.47	**0.081**

Data expressed as mean ± 1SD or as median (interquartile range)

Bold highlights the *p* values that are statistically significant

*DWI* Diffusion Weighted Imaging, *ADC* Apparent Diffusion Coefficient, *FA* Fractional Anisotropy, *NAA* N-acetylaspartate, *Cr* creatine, *Cho* choline, *mI myo*-inositol, Glx-glutamine/glutamate

#### Diffusion weighted imaging

The median ADC in the body of the corpus callosum was increased in the patients with cirrhosis, compared to healthy controls, but the difference was not significant (Table 1). The median ADCs in the two other studied regions of the corpus callosum were comparable between the two groups (Table 1). There were no significant differences in median ADC in relation to neuropsychiatric status.

There were no significant differences in the mean FA in the three regions of the corpus callosum between patients and controls (Table 1). There were no significant differences in mean FA in relation to neuropsychiatric status.

#### Magnetic resonance spectroscopy

There were no significant differences in mean basal ganglia metabolite ratios between patients and controls (Table 1).

### Correlation analysis

Significant positive correlations were observed between MTRs in the ROIs (*r* = 0.823 (putamen v globus pallidus); *r* = 0.720 (putamen v frontal white matter), *p* < 0.001 for both) but otherwise there were no significant correlations between ADC, MTR and spectroscopic intensities following correction for multiple comparison (Supplementary Figure and Table).

### Psychometric testing following LOLA

Psychometric performance, assessed using the PHES battery, improved in four of the seven patients with minimal HE following LOLA. No changes were observed in psychometric performance in the remaining patients.

### MR variables following LOLA

Follow-up MR scans were undertaken in eight of the 22 healthy controls. No differences were observed in any of the measured MR variables.

#### Brain volume

Twenty pairs of pre- and post-LOLA T_1_ brain volume datasets were available for analysis. There was insufficient contrast in the caudal regions of the brains in the remaining two datasets which would have resulted in unreliable tissue segmentation/edge-finding. There was no differential change in global or regional brain volume in relation to or neuropsychiatric status or LOLA responsiveness.

#### Magnetization transfer ratios

There were no significant differences in median cerebral MTRs following LOLA in the patient group as a whole (Table 2). No post-LOLA differences were observed in MTRs in the patients by neuropsychiatric status or LOLA responsiveness.

**Table 2:** Regional cerebral magnetization transfer ratios in patients with cirrhosis pre- and post-LOLA

Brain region	Magnetization Transfer Ratio		Significance (*p*)
	Pre-LOLA (*n* = 22)	Post-LOLA (*n* = 22)	
Frontal WM	57.0 (55.7–57.4)	56.5 (56.0–57.3)	0.85
Caudate	46.8 (45.7–47.6)	46.1 (45.6–47.7)	0.44
Putamen	48.2 (47.3–48.9)	47.8 (47.3–49.1)	0.95
Globus pallidus	51.4 (50.5–52.6)	51.5 (50.1–52.0)	0.58
Thalamus	51.5 (50.5–51.9)	51.2 (50.4–51.8)	0.48

Data expressed as median (interquartile range)

*WM* white matter

#### Diffusion weighted imaging

There was no significant difference in mean cerebral ADCs or mean cerebral FA in patients following LOLA, either in the group as a whole (Table 3) or in the subgroups defined by neuropsychiatric status or LOLA responsiveness.

**Table 3:** Cerebral diffusion weighted imaging variables in the corpus callosum in patients with cirrhosis pre- and post-LOLA

Brain Region	Patients with Cirrhosis		Significance (*p*)
	Pre-LOLA (*n* = 21)	Post LOLA (*n* = 22)	
Apparent Diffusion Coefficient (10^–3^ mm^2^/s)			
Genu	0.82 (0.81–0.84)	0.83 (0.79–0.85)	0.93
Splenium	0.72 (0.70–0.77)	0.73 (0.70–0.76)	0.77
Body	0.83 (0.80–0.84)	0.81 (0.80–0.84)	0.37
Functional Anisotropy (0–1)			
Genu	0.75 ± 0.03	0.74 ± 0.05	0.35
Splenium	0.85 ± 0.04	0.84 ± 0.05	0.99
Body	0.75 ± 0.04	0.74 ± 0.04	0.31

Data expressed as median (interquartile range) or mean ± 1SD

#### Magnetic resonance spectroscopy

The coefficient of variation for NAA/Cr was 0.041 with a variability of 1 % between the first and tenth scans. The coefficient of variation for Cho/Cr was 0.098 with a variability of 4.35 %. There was no significant difference in the measured basal ganglia metabolite ratios in the patients with cirrhosis following LOLA or in sub-groups of patients defined by neuropsychiatric status or LOLA responsiveness. There were no significant associations between the MRS data derived from the putamen, globus pallidus and thalamus and the Cho/Cr in the basal ganglia.

## Discussion

This study was designed to investigate whether measures of cerebral volume, water content and metabolite concentrations, obtained using cerebral MR imaging and spectroscopy, could be used to differentiate patients with minimal HE from their neuropsychiatrically unimpaired counterparts and/or to monitor the effects of measures designed to change neuropsychiatric status*.* LOLA, an ammonia–lowering agent, which has been used to treat HE (Fleig et al. 1999; Poo et al. 2006; Stauch et al. 1998), was given to all patients, irrespective of neuropsychiatric status, because the purpose of the study was not to demonstrate the efficacy of this agent as a treatment, but to use it as a therapeutic tool to effect change in psychometric performance, and thus determine if the MR sequences under investigation were sufficiently sensitive to detect change within the brain that might correlate with the psychometric changes. Good quality cerebral MR data were obtained, using a 3 T system, from well-characterised populations of patients with cirrhosis and matched healthy controls. However, no definitive baseline MR features were observed that either distinguished the patients from the controls, or effectively differentiated the patients with minimal HE from those who were neuropsychiatrically unimpaired. Four of the seven subjects (50 %) classified as having minimal HE, at baseline, showed improvement in psychometric performance, to within the normal range, following 4 weeks of LOLA, but this was not accompanied by any significant changes in MR variables overall.

The significant positive correlations observed between regional cerebral MTRs values suggest that the abnormalities of water distribution are widespread. Biochemical and white matter (tractography) abnormalities are not well correlated suggesting that the more functional aspects of interconnectivity may be more localized. This is supported by previous finding in this population in relation to the default mode network (McPhail et al. 2013).

One of the limitations of the study is the relatively small number of patients with minimal HE. This was undoubtedly a feature of the recruitment procedures as consecutive eligible patients, with no evidence of clinical HE, were selected from amongst ‘well’ out-patients with cirrhosis and they only underwent psychometric testing once selected. However, patients with cirrhosis with possible confounding or co-occurring condition which might affect their cerebral function were excluded and the study was controlled.

The results of the present study both support and contradict previous findings. Thus, in this study mean MTRs were significantly reduced in the frontal white matter, globus pallidus and thalamus in patients with cirrhosis, compared to healthy controls and were lower in the frontal white matter in the patients with minimal HE, compared to the controls but not to their unimpaired counterparts. In addition, there was no significant difference between MTRs before and after LOLA, even in those patients in whom psychometric performance improved.

Other studies have demonstrated reversibility of cerebral MTRs after liver transplantation (Córdoba et al. 2001) and after treatment with rifaximin and lactulose (Rai et al. 2015). However, in the present study, both the level of psychometric impairment and severity of liver disease were subtler than in previous studies. Thus, the magnitude of baseline abnormalities may be less pronounced compared to those in patients with more severe liver disease. Subsequently, the ability to detect a statistically significant change in, for example, MTR following an improvement in psychometric performance may be more limited.

A 15-direction DWI sequence was used for data acquisition in the present study, with ROIs in three regions of the corpus callosum. These measurements demonstrated increased median ADCs in the body of the corpus callosum in patients with cirrhosis, compared to healthy controls, but this did not reach statistical significance after correction for multiple comparisons. An increase in ADCs may result from minimal cellular oedema with an increase of membrane permeability and increased intracellular diffusivity, as well as changes in the viscosity of the cytoplasm (Lodi et al. 2004). The findings in the present study contrast with those of Sugimoto and colleagues who studied 24 healthy controls of mean age 62 ± 9 years and 10 subjects with cirrhosis and minimal HE of mean age 70 ± 6 years, the majority of whom had moderately decompensated disease (Sugimoto et al. 2008). They reported increased ADCs in the frontal and parietal white matter, but found no statistically significant difference in the putamen, globus pallidus or cingulate regions.

In the present study, there was no significant change in median regional ADCs before and after oral LOLA in the whole patient cohort or the sub-category of responders to LOLA. This is not surprising, given the fact that there were no differences in baseline ADC values between patients and controls. However, these findings contrast with those of Kale et al. (2006) who reported a significant reduction in ADCs in the corpus callosum, right internal capsule, left internal capsule, caudate nucleus, putamen, frontal and occipital white matter in 10 patients with minimal HE treated with lactulose for a total of 3 weeks. The functional severity of the liver disease in this subgroup of 10 patients was not specified, but the majority of the original cohort of 39 patients had decompensated disease.

There were no significant differences in mean baseline cerebral FA between healthy controls and the patients with cirrhosis in the present study and there were no changes following LOLA, even in those who responded with an improvement in psychometric performance. Ahluwalia and colleagues reported significant increases in mean cerebral FA in 16 patients with minimal HE following 8 weeks of treatment with rifaximin, during which time psychometric performance improved significantly (Ahluwalia et al. 2014). However, the interpretation of these results is confounded because no healthy control data were provided. Chen and colleagues studied 65 patients with cirrhosis, 29 of whom had minimal HE, while the remaining 36 had no evidence of neuropsychiatric impairment (Chen et al. 2015). The mean FA was significantly reduced in multiple cerebral regions and the changes in FA values were found, after adjustment for the degree of hepatic dysfunction, to be predictive of survival over the next 18 months. Again, the absence of a healthy control group hampers the interpretation of the data. Finally, Lin and colleagues found no significant changes in mean FA in 28 liver transplant candidates with minimal HE, compared to 30 healthy, matched controls (Lin et al. 2014). However, studies repeated 6 to 12 months following liver transplantation showed a decrease in FA in the right temporal lobe which, along with the changes observed in other imaging modalities were attributed to demyelination and gliosis.

The overall interpretation of the DWI findings in the present study and in the others undertaken to date is difficult. Study protocols differ appreciably and so do the methods of data analysis and the variables selected for study; thus direct comparisons are difficult, if not impossible to make. Chen and colleagues, for example, interpreted the increase they observed in cerebral mean diffusivity (MD), in patients with minimal HE, as indicating intracerebral oedema and the reduction in cerebral FA as reflecting impaired microstructural integrity, but without further elaboration (Chen et al. 2015). Ahluwalia and colleagues interpreted their findings, in patients with minimal HE, of an increase in cerebral FA following treatment with rifaximin in the absence of any change in cerebral MD as indicative of a treatment–related correction of intracellular oedema (Ahluwalia et al. 2014). Finally, Lin and colleagues interpreted the trend to an increase in cerebral MD that they observed in patients with minimal HE, but with no change in cerebral FA as perhaps indicating the co-existence of extracellular and intracellular oedema (Lin et al. 2014). The findings in the present study could be similarly explained.

^1^H MRS in patients with cirrhosis and minimal HE is normally typified by the finding of increased cerebral Glx/Cr and reduced mI/Cr and Cho/Cr ratios (Córdoba et al. 2001; Miese et al. 2006; Taylor-Robinson et al. 1996; Meng et al. 2015). The choline resonance contains components from compounds that participate in phospholipid metabolism and osmotic regulation in glial cells. The reduction in the Cho/Cr in patients with cirrhosis is considered to represent osmolar changes in the brain, whereas an elevated Cho/Cr is considered to represent myelin destruction, increased membrane synthesis or neuroinflammation (Patel et al. 2012). mI is a sugar involved in the synthesis of phosphoinositides for cerebral osmoregulation. The main reservoir of mI is within the glial cells, thus it is proposed to be a marker of glial cell osmoregulation. (Patel et al. 2012).

The observation that the mean cerebral metabolite ratios were unchanged in the patients with cirrhosis, in the present study, was unexpected. This finding may reflect differences in the MR scanning parameters and the selected ROIs between studies. Thus, the present study was undertaken at a magnetic field strength of 3 T whereas most previous studies have been conducted at 1.5 T. As MR visibility is sequence dependent it was anticipated that with the inherent signal to noise advantage of imaging at 3 T, it might be possible to better differentiate and quantify individual metabolite peaks. Similarly, previous studies have provided MRS information from areas of the brain such as the parieto-occipital cortex (Córdoba et al. 2001, 2002), deep white matter (Häussinger et al. 1994) and the visual cortex (Laubenberger et al. 1997) whereas in this study MRS information was provided from the basal ganglia, where susceptibility effects related to manganese deposition may have affected tissue properties resulting in changes in relaxation times and ultimately on Cho/Cr or mI/Cr ratios. This effect may be even more apparent at 3 T (Ochi et al. 2011).

In conclusion: changes in cerebral MTR are observed in patient with cirrhosis, but with the exception of a reduction in MTR in the frontal region none was specific for the presence of minimal HE. Although four of seven patients with minimal HE showed improvement in psychometric testing following LOLA this was not reflected in any discernable changes in MR variables. Based on this study it would not appear that cerebral MRI or MRS have sufficient utility for the diagnosis of minimal HE or the monitoring of treatment effects in this patient group to recommend they use in routine clinical practice. Justification for use of these techniques in a research setting should to be based on proof of diagnostic efficacy obtained from a large multicenter trial utilizing strict protocol standardization for MR data acquisition.

## Electronic supplementary material

ESM 1 (DOCX 22 kb)

ESM 2 (DOCX 19 kb)
