# Supplementary material for: A longitudinal study of patients with cirrhosis treated with L-ornithine L-aspartate, examined with magnetization transfer, diffusion-weighted imaging and magnetic resonance spectroscopy
Source: Metab Brain Dis. 2016 Aug 3;32(1):77–86. doi: 10.1007/s11011-016-9881-3 (PMC5225223; doi:10.1007/s11011-016-9881-3)
Supplement: Supplementary file 1 — (DOCX 22 kb) [file 11011_2016_9881_MOESM1_ESM.docx]

**Correlation table**

|  | | ADC Body | ADC Genu | ADC splenium | Cho/Cr | mI/Cr | MTR Caudate | MTR Frontal | MTR Globus | MTR Putamen | MTR Thalamus | NAA/Cr |
| --- | --- | --- | --- | --- | --- | --- | --- | --- | --- | --- | --- | --- |
| ADC Body | Correlation coefficient Significance Level P n |  | 0.173 0.4661 20 | 0.270 0.2490 20 | 0.335 0.1614 19 | 0.036 0.8923 17 | -0.044 0.8554 20 | 0.055 0.8163 20 | 0.205 0.3860 20 | 0.078 0.7443 20 | 0.169 0.4754 20 | 0.352 0.1389 19 |
| ADC Genu | Correlation coefficient Significance Level P n | 0.173 0.4661 20 |  | 0.194 0.4130 20 | 0.441 0.0585 19 | -0.078 0.7664 17 | -0.028 0.9063 20 | 0.026 0.9146 20 | 0.247 0.2944 20 | 0.208 0.3792 20 | 0.288 0.2174 20 | 0.404 0.0866 19 |
| ADC splenium | Correlation coefficient Significance Level P n | 0.270 0.2490 20 | 0.194 0.4130 20 |  | -0.032 0.8968 19 | -0.130 0.6191 17 | 0.114 0.6336 20 | 0.196 0.4072 20 | 0.422 0.0639 20 | 0.390 0.0894 20 | 0.160 0.5005 20 | 0.015 0.9503 19 |
| Cho/Cr | Correlation coefficient Significance Level P n | 0.335 0.1614 19 | 0.441 0.0585 19 | -0.032 0.8968 19 |  | 0.289 0.2598 17 | 0.079 0.7469 19 | 0.281 0.2436 19 | 0.259 0.2848 19 | 0.305 0.2042 19 | 0.402 0.0882 19 | 0.253 0.2957 19 |
| mI/Cr | Correlation coefficient Significance Level P n | 0.036 0.8923 17 | -0.078 0.7664 17 | -0.130 0.6191 17 | 0.289 0.2598 17 |  | 0.300 0.2428 17 | 0.116 0.6565 17 | 0.026 0.9226 17 | -0.025 0.9240 17 | -0.168 0.5191 17 | -0.154 0.5547 17 |
| MTR Caudate | Correlation coefficient Significance Level P n | -0.044 0.8554 20 | -0.028 0.9063 20 | 0.114 0.6336 20 | 0.079 0.7469 19 | 0.300 0.2428 17 |  | 0.635 0.0020 21 | 0.539 0.0116 21 | 0.609 0.0034 21 | 0.355 0.1144 21 | -0.167 0.4954 19 |
| MTR Frotnal | Correlation coefficient Significance Level P n | 0.055 0.8163 20 | 0.026 0.9146 20 | 0.196 0.4072 20 | 0.281 0.2436 19 | 0.116 0.6565 17 | 0.635 0.0020 21 |  | 0.742 0.0001 21 | 0.720 0.0002 21 | 0.640 0.0018 21 | 0.067 0.7842 19 |
| MTR Globus | Correlation coefficient Significance Level P n | 0.205 0.3860 20 | 0.247 0.2944 20 | 0.422 0.0639 20 | 0.259 0.2848 19 | 0.026 0.9226 17 | 0.539 0.0116 21 | 0.742 0.0001 21 |  | 0.823 <0.0001 21 | 0.437 0.0478 21 | 0.107 0.6629 19 |
| MTR Putamen | Correlation coefficient Significance Level P n | 0.078 0.7443 20 | 0.208 0.3792 20 | 0.390 0.0894 20 | 0.305 0.2042 19 | -0.025 0.9240 17 | 0.609 0.0034 21 | 0.720 0.0002 21 | 0.823 <0.0001 21 |  | 0.502 0.0203 21 | 0.003 0.9911 19 |
| MTR Thalamus | Correlation coefficient Significance Level P n | 0.169 0.4754 20 | 0.288 0.2174 20 | 0.160 0.5005 20 | 0.402 0.0882 19 | -0.168 0.5191 17 | 0.355 0.1144 21 | 0.640 0.0018 21 | 0.437 0.0478 21 | 0.502 0.0203 21 |  | 0.197 0.4198 19 |
| NAA/Cr | Correlation coefficient Significance Level P n | 0.352 0.1389 19 | 0.404 0.0866 19 | 0.015 0.9503 19 | 0.253 0.2957 19 | -0.154 0.5547 17 | -0.167 0.4954 19 | 0.067 0.7842 19 | 0.107 0.6629 19 | 0.003 0.9911 19 | 0.197 0.4198 19 |  |

Pearson correlation coefficient

Table 1: Correlation coefficients among MR measured parameters. To avoid false positives an FDR correction p value of <0.005 is required for significance. The table is ordered alphabetically (ADC – apparent diffusion coefficient, MTR – magnetization transfer ratios , Cho – choline, Cr- creatinine, NAA – N acetylaspartate, mI – myoinositol)
