# Supplementary material for: A longitudinal study of patients with cirrhosis treated with L-ornithine L-aspartate, examined with magnetization transfer, diffusion-weighted imaging and magnetic resonance spectroscopy
Source: Metab Brain Dis. 2016 Aug 3;32(1):77–86. doi: 10.1007/s11011-016-9881-3 (PMC5225223; doi:10.1007/s11011-016-9881-3)
Supplement: Supplementary file 2 — (DOCX 19 kb) [file 11011_2016_9881_MOESM2_ESM.docx]

|  |
| --- |
| \| MTR Putamen \|  \| 0.823 \| 0.720 \| 0.502 \| 0.609 \| 0.305 \| 0.208 \| 0.390 \| 0.078 \| 0.003 \| -0.025 \| \| --- \| --- \| --- \| --- \| --- \| --- \| --- \| --- \| --- \| --- \| --- \| --- \| \| MTR Globus \| 0.823 \|  \| 0.742 \| 0.437 \| 0.539 \| 0.259 \| 0.247 \| 0.422 \| 0.205 \| 0.107 \| 0.026 \| \| MTR Frontal \| 0.720 \| 0.742 \|  \| 0.640 \| 0.635 \| 0.281 \| 0.026 \| 0.196 \| 0.055 \| 0.067 \| 0.116 \| \| MTR Thalamus \| 0.502 \| 0.437 \| 0.640 \|  \| 0.355 \| 0.402 \| 0.288 \| 0.160 \| 0.169 \| 0.197 \| -0.168 \| \| MTR Caudate \| 0.609 \| 0.539 \| 0.635 \| 0.355 \|  \| 0.079 \| -0.028 \| 0.114 \| -0.044 \| -0.167 \| 0.300 \| \| Cho/Cr \| 0.305 \| 0.259 \| 0.281 \| 0.402 \| 0.079 \|  \| 0.441 \| -0.032 \| 0.335 \| 0.253 \| 0.289 \| \| ADC Genu \| 0.208 \| 0.247 \| 0.026 \| 0.288 \| -0.028 \| 0.441 \|  \| 0.194 \| 0.173 \| 0.404 \| -0.078 \| \| ADC Splenium \| 0.390 \| 0.422 \| 0.196 \| 0.160 \| 0.114 \| -0.032 \| 0.194 \|  \| 0.270 \| 0.015 \| -0.130 \| \| ADC Body \| 0.078 \| 0.205 \| 0.055 \| 0.169 \| -0.044 \| 0.335 \| 0.173 \| 0.270 \|  \| 0.352 \| 0.036 \| \| NAA/Cr \| 0.003 \| 0.107 \| 0.067 \| 0.197 \| -0.167 \| 0.253 \| 0.404 \| 0.015 \| 0.352 \|  \| -0.154 \| \| mI/Cr \| -0.025 \| 0.026 \| 0.116 \| -0.168 \| 0.300 \| 0.289 \| -0.078 \| -0.130 \| 0.036 \| -0.154 \|  \| \|  \| MTR Putamen \| MTR  Globus \| MTR Frontal \| MTR Thalamus \| MTR Caudate \| Cho/Cr \| ADC Genu \| ADC Splenium \| ADC Body \| NAA/Cr \| mI/Cr \| |

Pearson correlation coefficient

Figure S1: Correlogram ordered by significance of correlation coefficient and coloured by red (strong positive correlation) to blue (strong negative correlation). Other than high positive and significant correlation between MTR in the four cerebral regions measured there are no major significant correlations between the MR variables. alphabetically (ADC – apparent diffusion coefficient, MTR – magnetization transfer ratios , Cho – choline, Cr- creatinine, NAA – N acetylaspartate, mI – myoinositol)
